# High-risk human papillomavirus infection and cervical cytopathology: relationship with cervical nitric oxide levels

**DOI:** 10.1186/s12985-024-02435-6

**Published:** 2024-08-02

**Authors:** Doaa Mahdy El-Wakil, Olfat G. Shaker, Ahmed S. S. A. Rashwan, Yasmine Fathy Elesawy, Nermin Samir

**Affiliations:** 1 Department of Medical Microbiology and Immunology, Faculty of Medicine, Cairo University, Al-Saray Street, Al-Manial, Cairo, 11562 Egypt; 2 Departmet of Medical Biochemistry and Molecular Biology, Faculty of Medicine, Cairo University, Cairo, Egypt; 3 Department of Obstetrics and Gynecology, Faculty of Medicine, Cairo University, Cairo, Egypt; 4 Department of Anatomical Pathology, Faculty of Medicine, Cairo University, Cairo, Egypt

**Keywords:** High-risk human papillomavirus (hrHPV), Genotypes, Cytopathology, Atypia, Cervical nitric oxide

## Abstract

**Background:**

Nitric oxide (NO) may contribute to the persistence of high-risk human papillomavirus (hrHPV) infection, which has been linked to the development of premalignant lesions and cervical cancer. Our study aimed to examine the relationship between cervical NO metabolite (NOx) levels, hrHPV infection, and cytopathological findings. Additionally, we assessed cervical NOx levels as a biomarker for predicting hrHPV infection and epithelial atypia.

**Methods:**

The study involved 74 women who attended the Gynecology and Obstetrics outpatient clinics at Cairo University Hospitals between November 2021 and August 2022. Cervical samples were subjected to Pap testing, assessment of NOx levels by the Griess method, and detection of hrHPV DNA by real-time polymerase chain reaction.

**Results:**

High-risk HPV was detected in 37.8% of women. EA was found in 17.1% of cases, with a higher percentage among hrHPV-positive than negative cases (35.7% vs. 4.3%, *p* = 0.001). The most prevalent hrHPV genotype was HPV 16 (89.3%). The cervical NOx level in hrHPV-positive cases was significantly higher (37.4 µmol/mL, IQR: 34.5–45.8) compared to negative cases (2.3 µmol/mL, IQR: 1.2–9.8) (*p* = < 0.001). Patients with high-grade atypia showed significantly higher NOx levels (38.0 µmol/mL, IQR: 24.6–94.7) in comparison to NILM and low-grade atypia cases (5.0 µmol/mL, IQR: 1.6–33.3 and 34.5 µmol/mL, IQR: 11.7–61.7, respectively) (*p* = 0.006). Although the NOx levels among hrHPV-positive cases with low-grade atypia (40.4 µmol/mL, IQR: 33.3‒61.8) were higher than those with NILM (36.2 µmol/mL, IQR: 35.7‒44.0) and high-grade atypia (38.0 µmol/mL, IQR: 24.6‒94.7), the difference was not significant (*p* = 0.771). ROC curve analysis indicated that the cervical NOx cut-off values of > 23.61 µmol/mL and > 11.35 µmol/mL exhibited good diagnostic accuracy for the prediction of hrHPV infection and EA, respectively.

**Conclusions:**

The high prevalence of hrHPV infection, particularly HPV 16, in our hospital warrants targeted treatment and comprehensive screening. Elevated cervical NOx levels are associated with hrHPV infection and high-grade atypia, suggesting their potential use as biomarkers for predicting the presence of hrHPV and abnormal cytological changes.

## Introduction

Cervical cancer ranks as the eighth most common malignancy among women worldwide [1]. The International Agency for Research on Cancer (IARC) identifies 12 high-risk human papillomavirus (hrHPV) types that significantly contribute to cervical cancer development [2]. It has been estimated that approximately 71% of new cervical cancer cases are attributed to HPV 16 and HPV 18, whereas an additional 19% of cases are associated with HPV types 31, 33, 45, 52, and 58 [3].

Persistent hrHPV infection has been recognized as the leading cause of precancerous and malignant cervical lesions. Chronic inflammation and oxidative stress (OS) caused by persistent hrHPV infection play a pivotal role in the carcinogenic process [4]. Chronic inflammation impairs cell homeostasis, affecting normal cell growth and eventually malignant transformation. Additionally, it leads to OS, where the production of reactive oxygen and nitrogen species (RONS) is increased promoting DNA damage, thereby inducing mutations and genomic alterations [5]. Moreover, OS can be generated by the early expressed hrHPV proteins by decreasing the expression and activity of antioxidants, resulting in DNA damage that subsequently induces the integration of the viral genome into the cellular one, triggering the carcinogenic process [6].

Nitric oxide (NO) is a free radical gas that belongs to RONS and is considered a vital cofactor in cervical carcinogenesis induced by chronic HPV infection [7]. Several risk factors have been found to increase cervical NO levels, such as smoking, multiparity, prolonged use of oral contraceptives, concurrent sexually transmitted infections, and chronic inflammation [8]. This increase in NO results in decreased p53 and pRb levels and reduced apoptotic activity in HPV-infected cervical cells, hence increasing the survival of mutant cells resulting in oncogenesis [9]. Noteworthy, it has been shown that HPV infection is associated with elevated levels of NO metabolites (NOx) in vitro [10] and in vivo [11], and these high levels may facilitate the persistence of hrHPV infection. The underlying mechanisms are ambiguous but could be related to the NO ability to stimulate the transcription of HPV oncoproteins that maintain HPV persistence [9, 12].

Our study aimed to detect the prevalence, genotypes, and significance of hrHPV infection in relation to clinical and cytopathological findings. We also aimed to assess the cervical NOx levels among women with and without hrHPV infection, as well as those with normal and abnormal Papanicolaou (Pap) tests. Additionally, we attempt to determine whether cervical NO levels could be used as a biomarker for diagnosing patients with hrHPV infection and abnormal cytopathological changes.

## Subjects and methods

### Population and setting of the study

This cross-sectional analytical study was conducted over 10 months, from November 2021 through August 2022, and included 74 non-pregnant women. Sixty-three women attended the Kasr Al-Ainy outpatient clinics of Gynecology and Obstetrics at Cairo University Hospitals for gynecological examination, and 11 patients with a history of abnormal Pap smear underwent conventional colposcopy examination at Kasr Al-Ainy Colposcopy Unit. Patients with local cervical bleeding, previous malignancy, previously received HPV vaccines, or those with a history of prior therapy, such as hysterectomy, trachelectomy, chemotherapy, or radiotherapy were excluded from the study. History was taken from each patient, including parity, smoking, marital status, sexual partners, previous Pap tests, contraceptive method (oral, intrauterine device (IUD), or others) as well as cervical gynecological examination. The study was approved by the Research Ethics Committee of the Institutional Review Board, Faculty of Medicine, Cairo University, Egypt (ID: N-89-2021). Written informed consent was obtained from each woman before the study. This research was conducted according to the guidelines outlined by the Declaration of Helsinki.

### Specimen collection and transport

Three samples were collected from each participant in the study. Cervical Pap smear sample was fixed in 95% ethyl alcohol. Pap staining and cytological examination were performed in the Cytopathology Unit, Pathology Department, Faculty of Medicine, Cairo University. Two cervical fluid samples were collected, one sample was transported to the Medical Biochemistry and Molecular Biology Department, Faculty of Medicine, Cairo University for NOx levels detection and the other to the Medical Microbiology and Immunology Department, Faculty of Medicine, Cairo University for hrHPV DNA detection.

### Pap smear testing

Cervical sampling for the Pap test was collected before any NOx and HPV detection sampling to ensure adequate pap smear cellularity. Conventional pap smears were obtained using a modified Ayer’s plastic spatula for sampling the cervix and transformation zone using an endocervical brush. The samples were evenly smeared on labeled slides with the patient’s code and immediate fixation was done to prevent air-drying artifacts [13]. Cervical cytology results were interpreted according to the terminology of The Bethesda System (TBS) for reporting cervical cytopathology, including the criteria for sample adequacy [14]. The assessment of evident inflammation and reparative cellular changes was performed according to the detailed cytopathologic criteria previously described. Marked inflammation was documented when inflammatory cells were observed attacking the epithelial cells with cytolysis and granular debris background. Reparative cellular changes included the presence of perinuclear halo, conspicuous nucleoli, cytoplasmic fraying, and polychromasia [15].

The high-grade atypia cases were referred for colposcopy and tissue biopsy. One studied LSIL case in cytology has coincidently performed a total hysterectomy for dysfunctional uterine bleeding and leiomyomas. Hematoxylin and Eosin (H&E) stained tissue sample slides were examined and epithelial dysplasia was evaluated. Immunohistochemical tests were performed for Ki67 (rabbit polyclonal antibody, Cat. #RB-9043-P; 1:300) and p16 (mouse monoclonal antibody, clone 16P04, 1:40) (Lab Vision, Fremont, CA, USA) using immunohistochemistry autostainer (BenchMark ULTRA, Ventana, Arizona, USA). Sections of vaginal squamous cell carcinoma and reactive lymph node were used as the positive control for p16 and ki67, respectively. Negative controls were done by replacing primary antibodies with buffer. Nuclear staining for ki67 as well as both cytoplasmic and nuclear staining for p16 were considered as the positive stain. Pap smears, H&E, and immunohistochemically stained slides were evaluated by Leica light microscopy.

### Detection of NOx levels in cervical secretions by the Griess method

Assessment of NOx levels in cervical fluid samples was done as described previously [16]. Dacron swab (Titan Biotech Ltd, Bhiwadi, India) was kept in the cervical canal for 20 s., washed in 1.5 ml physiological saline solution (HiMedia, Mumbai, India), then stored at − 20 °C for less than 3 months until analyzed. Following the manufacturer’s instructions, the samples were further examined for nitrite metabolites by the microplate assay using the Griess method (Invitrogen, Thermo Fisher Scientific, USA). In 96-well flat-bottomed sterile polystyrene plates (Nunc, Roskilde, Denmark), 150 µL of the sample or calibrators was mixed with 20 µL of Griess Reagent and 130 µL of deionized water. The mixture was incubated for 30 min. at room temperature, and the absorbance was measured at 548 nm using the Sunrise Tecan plate reader (TECAN, Switzerland). The calibrators were prepared by serial dilution of the nitrite standard solution provided by the kit with deionized water to achieve concentrations between 1 and 100 µmol/mL. A standard curve was plotted, and the concentrations of samples were calculated. The detection limit of this method for the detection of NOx level was 1.0 µmol/mL. The samples and calibrators were tested in triplicate and the absorbance values were averaged.

### Molecular detection and genotyping of hrHPV

Cervical fluid samples were collected and transported using a viral transport medium (Titan Biotech Ltd, Bhiwadi, India**)**, then kept at − 70 °C until analyzed for the presence of hrHPV. The HPV DNA was extracted using QIAamp DNA Mini kit (Qiagen, GmbH, Germany) following the manufacturer’s guidelines. The extracted DNA was subjected to real-time polymerase chain reaction (PCR) for determination of hrHPV genotypes 16, 18, 31, 33, 35, 39, 45, 51, 52, 56, 58, 59, 66, and 68 using a commercially available kit (PrimerDesign Ltd., Southampton, Hants, UK). This kit allows for the detection of the most prevalent hrHPV-16 and 18 genotypes on different filters (ROX and VIC, respectively), while the remaining genotypes were interpreted together on a single filter (FAM), making it unable to distinguish between them. Amplification was performed in a Rotor-Gene Q MDX instrument with software version 2.3.3 (Qiagen, Germany). The PCR mixture included 10 µL of the oasig master mix (PrimerDesign Ltd., Southampton, Hants, UK), 1 µL of hrHPV primer/probe mix, 5 µL of the DNA template, and finally 4 µL RNase/DNase free water to complete a total reaction volume of 20 µL per well. The thermocycling conditions were as follows: an initial enzyme activation step at 95 ^o^C for 2 min., followed by 50 cycles of denaturation step at 95 ^o^C for 10 s. and data collection at 60 ^o^C for 60 s. Fluorescence was read in ROX, FAM, VIC, and Cy5 channels. High-risk HPV templates containing the 14 genotypes were the positive control, and RNAse/DNAse-free water was the negative control, both were included in each PCR run.

### Statistical methods

Data were analyzed using IBM SPSS advanced statistics (Statistical Package for Social Sciences), version 27 (SPSS Inc., Chicago, IL). Numerical data were expressed in mean and standard deviation or median and interquartile range (IQR), while categorical data were described as numbers and percentages. Comparisons between the two groups were done using the Student’s t-test for normally distributed numeric variables, and the Mann-Whitney *U* test for non-normally distributed numeric variables. Comparisons between the three groups were conducted using the Kruskal-Wallis test. For categorical variables, comparisons were performed using the chi-square test or Fisher’s exact test as appropriate. Receiver-operating characteristic (ROC) curve analysis was done to determine the optimal cut-off value of cervical NOx levels that are associated with Pap atypia and the presence of hrHPV. ROC curves were created by calculating the sensitivities and specificities of cervical fluid NOx levels at several cut-off points. A *p*-value of ≤ 0.05 was considered statistically significant.

## Results

### Demographics and clinical characteristics of the studied patients

This study was conducted on 74 women seeking gynecologic consultation in Gynecology and Obstetrics outpatient clinics at Cairo University Hospitals. Their age ranged from 20 to 64 years (mean: 38.7 ± 10.9 years). High-risk HPV was detected in 37.8% (28/74) of the studied women. The mean age of hrHPV-positive cases was lower than negative cases (36.5 ± 9.0 years vs. 40.0 ± 11.9 years), with no statistically significant difference (*p* = 0.181). High-risk HPV-positive cases were mostly encountered in the age group 30 − 45 years (17/28, 60.7%), while lower rates were observed in women younger than 30 years (28.6%) and older than 45 years (10.7%). Table 1 presents the demographic and clinical characteristics of the studied patients.

**Table 1:** Comparison between hrHPV-positive and negative cases in relation to the demographic and clinical variables

Clinical variables	Total *N* = 74 (%)	hrHPV-positive *N* = 28 (%)	hrHPV-negative *N* = 46 (%)	*p*-value^a^
**Age (mean ± SD)**	38.7 ± 10.9	36.5 ± 9.0	40.0 ± 11.9	0.181^b^
**Age groups**				
˂ 30	20 (27.0)	8 (28.6)	12 (26.1)	0.134
30 − 45	37 (50.0)	17 (60.7)	20 (43.5)	
> 45	17 (23.0)	3 (10.7)	14 (30.4)	
**Gravidity**				
Nulligravida	26 (35.1)	7 (25.0)	19 (41.3)	0.154
Multigravida	48 (64.9)	21 (75.0)	27 (58.7)	
**Parity**				
Nulliparous	26 (35.1)	7 (25.0)	19 (41.3)	0.154
Parous	48 (64.9)	21 (75.0)	27 (58.7)	
Contraception* **(*N* = 67)**				
Yes	16 (21.6)	11 (39.3)	5 (10.9)	**0.002**
No	51 (68.9)	13 (46.4)	38 (82.6)	
Contraceptive method* ( ***N*** ** = 67)**				
None	51 (68.9)	13 (46.4)	38 (82.6)	NA
Oral	2 (2.7)	2 (7.1)	0 (0.0)	
IUD	11 (14.9)	7 (25)	4 (8.7)	
Others**	3 (4.0)	2 (7.1)	1 (2.2)	
**Sexual partner**				
Yes***	49 (66.2)	20 (71.4)	29 (63.0)	0.460
No	25 (33.8)	8 (28.6)	17 (37.0)	
**History of genital warts**				
Yes	4 (5.4)	2 (7.1)	2 (4.3)	0.631^c^
No	70 (94.6)	26 (92.9)	44 (95.7)	
**History of abnormal PAP smear**				
Yes	8 (10.8)	4 (14.3)	4 (8.7)	0.461^c^
No	66 (89.2)	24 (85.7)	42 (91.3)	

*Abbreviations* IUD, intrauterine device; NA, not applicable

*Seven cases were excluded from the data analysis and considered missing data

**Other methods include implants and injectables in the form of Depot medroxyprogesterone acetate (DMPA)

***A single sexual partner

^a^Pearson Chi-square test except where specified

^b^Student’s t-test

^c^Fisher’s Exact test

Data are expressed in mean ± standard deviation or numbers (percentages)

A *p*-value ≤ 0.05 is considered significant

Forty-eight women were both multigravida and parous (64.9% each), 16 (21.6%) women used contraception, 49 (66.2%) women had a single sexual partner, and 8 (10.8%) women experienced a previous history of abnormal Pap smear findings. Among hrHPV-positive cases, 39.3% (11/28) of them used contraception compared to the negative cases (10.9%, 5/46) (*p* = 0.002) (Table 1). There were only four cases (5.4%) with a history of previous genital warts. Our PCR analysis revealed that two of these cases exhibited the presence of hrHPV genotype 16.

### Cytopathological findings of the studied population

**Fig. 1:**
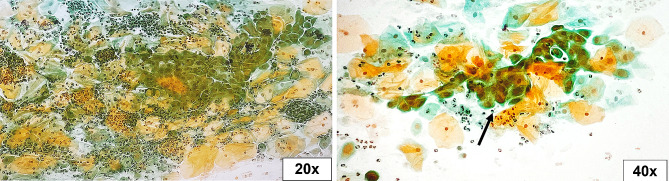
Atypical squamous cells of undetermined significance (ASC-US). Atypical repair cells (arrow) with focal enlarged subtle irregular nuclei among inflammation (Pap stain, 20x and 40x original magnifications)

**Fig. 2:**
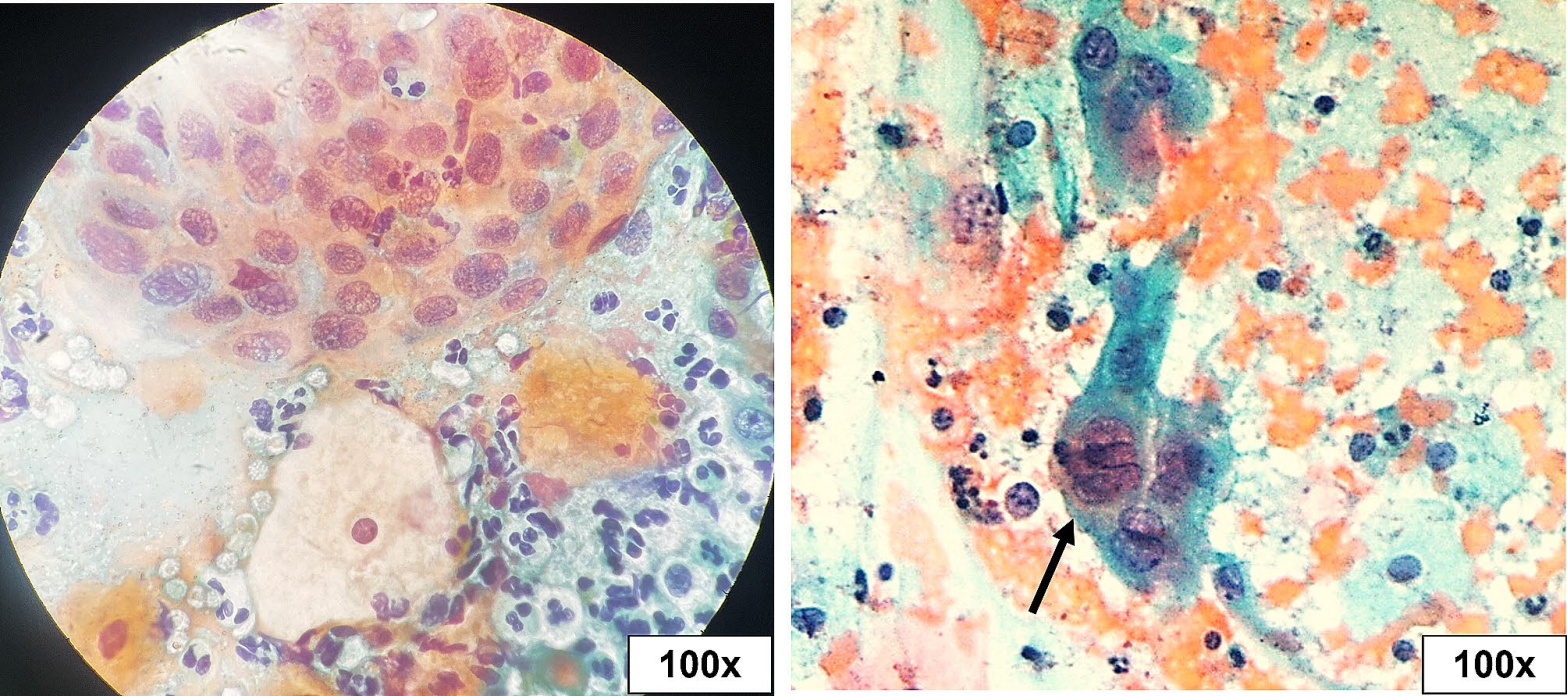
Atypical squamous cells - cannot exclude a high-grade squamous intraepithelial lesion (ASC-H). Few squamous cells with enlarged nuclei with multinucleation (arrow) in a dense hemorrhagic background (Pap stain, 100x original magnification)

**Fig. 3:**
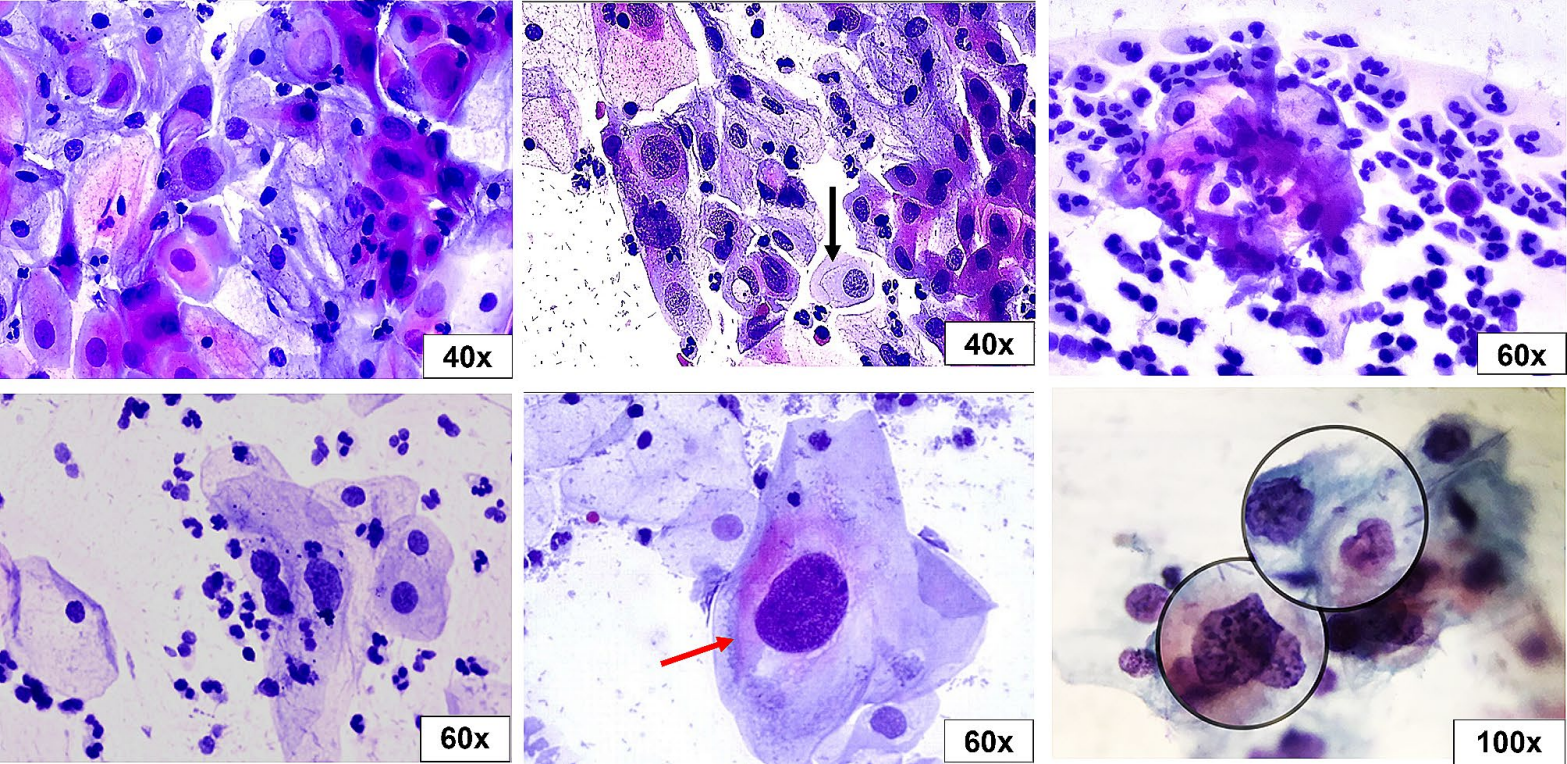
Low-grade squamous intraepithelial lesion (LSIL). Squamous cells showed enlarged nuclei (≥ 3x intermediate cell nucleus size), with punched-out cytoplasmic vacuolation (black arrow). Squamous cells showed enlarged nuclei, focal nuclear membrane irregularity, and multinucleation (circles) with megacyte (red arrow) (Pap stain, 40x, 60x, and 100x original magnifications)

**Fig. 4:**
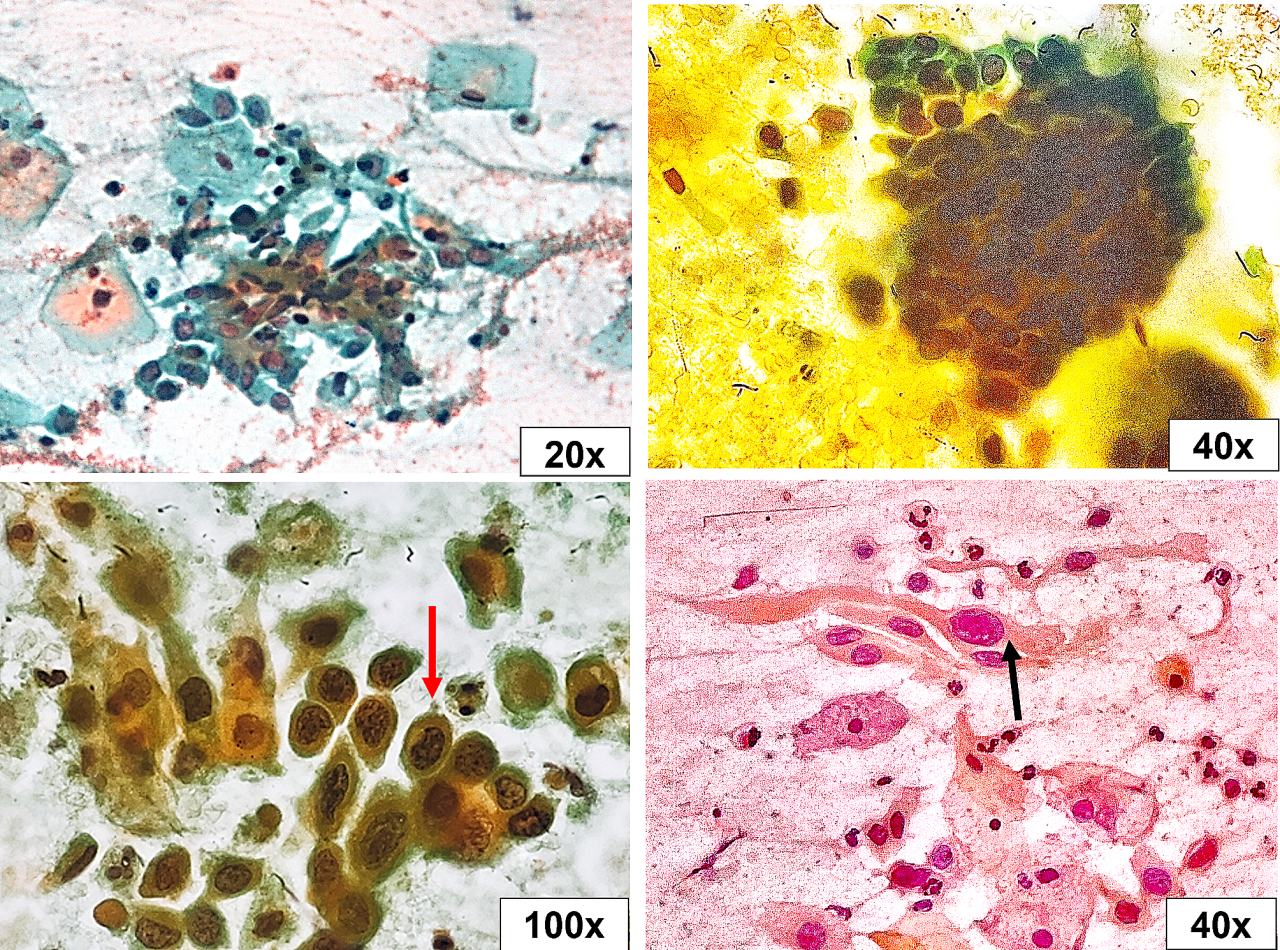
High-grade squamous intraepithelial lesion (HSIL). Parabasal cell size with enlarged hyperchromatic pleomorphic nuclei and evident nuclear membrane irregularities (red arrow). Tadpole cells (black arrow) were seen with cytoplasmic elongation and enlarged irregular nuclei (Pap stain, 20x, 40x, and 100x original magnifications)

**Fig. 5:**
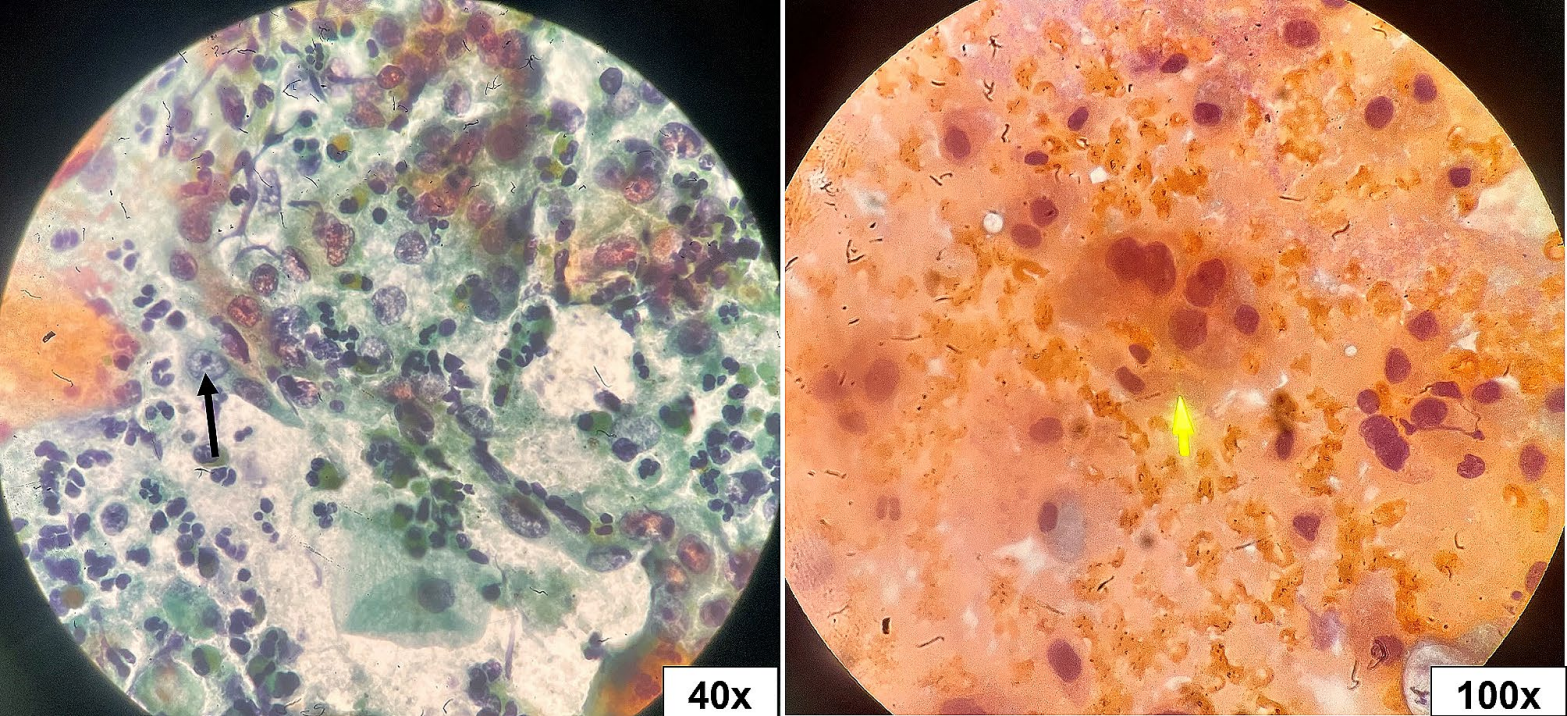
Atypical glandular cells (AGC). Endocervical cells (yellow arrow) with enlarged vesicular nuclei, multinucleation, and prominent nucleoli (black arrow) in a dense hemorrhagic background (Pap stain, 40x and 100x original magnifications)

**Fig. 6:**
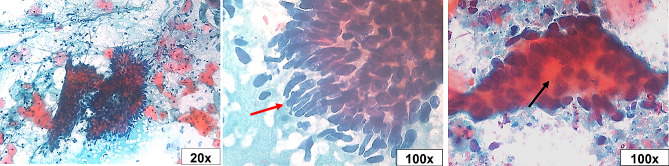
Adenocarcinoma in situ (AIS). Glandular endocervical cells with enlarged hobnailed nuclei (feathering) (red arrow) & acinar formation (black arrow) (Pap stain, 20x and 100x original magnifications)

The Pap smear was inadequate in 5.4% (4/74) of studied cases, of which two patients tested positive for hrHPV. Epithelial atypia (EA) was found in 17.1% of cases, while 82.9% of them fall under the negative for intraepithelial lesion or malignancy (NILM) category (Table 2). Epithelial atypia was discriminated according to TBS as follows: atypical squamous cells of undetermined significance (ASC-US) in four cases (5.7%), atypical squamous cells - cannot exclude a high-grade squamous intraepithelial lesion (ASC-H) in three cases (4.3%), low-grade squamous intraepithelial lesion (LSIL) in two cases (2.9%), high-grade squamous intraepithelial lesion (HSIL) in one case (1.4%), atypical glandular cells (AGC) in one case (1.4%), and adenocarcinoma in situ (AIS) in one case (1.4%) (Figs. 1, 2, 3, 4, 5, 6).

**Table 2:** Comparison between hrHPV-positive and negative cases in relation to the cytopathological results

Cytology	Total *N* = 74 (%)	hrHPV-positive *N* = 28 (%)	hrHPV-negative *N* = 46 (%)	*p*-value*
Bethesda grouping** ( ***N*** ** = 70)**				
NILM	58 (82.9)	16 (57.1)	42 (91.3)	**0.001**
EA	12 (17.1)	10 (35.7)	2 (4.3)	
Bethesda score** ( ***N*** ** = 70)**				
NILM	58 (82.9)	16 (57.1)	42 (91.3)	NA
ASC-US	4 (5.7)	2 (7.1)	2 (4.3)	
ASC-H	3 (4.3)	3 (10.7)	0 (0.0)	
LSIL	2 (2.9)	2 (7.1)	0 (0.0)	
HSIL	1 (1.4)	1 (3.6)	0 (0.0)	
AGC	1 (1.4)	1 (3.6)	0 (0.0)	
AIS	1 (1.4)	1 (3.6)	0 (0.0)	
Grade of EA** ( ***N*** ** = 70)**				
NILM	58 (82.9)	16 (57.1)	42 (91.3)	**< 0.001**
Low-grade	7 (10)	5 (17.9)	2 (4.3)	
High-grade	5 (7.1)	5 (17.9)	0 (0.0)	
Type of EA** ( ***N*** ** = 70)**				
NILM	58 (82.9)	16 (57.1)	42 (91.3)	**0.001**
Squamous	10 (14.3)	8 (28.6)	2 (4.3)	
Glandular	2 (2.8)	2 (7.1)	0 (0.0)	
**Biopsy results (** ***N*** ** = 4)**				
Dysplasia (CIN III)	2 (50)	2 (7.1)	0 (0.0)	NA
Carcinoma	2 (50)	2 (7.1)	0 (0.0)	
**Inflammation**				
Yes	55 (74.3)	20 (71.4)	35 (76.1)	0.656
No	19 (25.7)	8 (28.6)	11 (23.9)	
**Inflammation (** ***N*** ** = 55)**				
Mild	29 (52.7)	7 (25)	22 (47.8)	**0.047**
Marked	26 (47.3)	13 (46.4)	13 (28.3)	
**Reactive cells**				
Yes	27 (36.5)	13 (46.4)	14 (30.4)	0.166
No	47 (63.5)	15 (53.6)	32 (69.6)	

*Abbreviations* NILM, negative for intraepithelial lesion or malignancy; EA, epithelial atypia; ASC-US, atypical squamous cells of undetermined significance; ASC-H, atypical squamous cells cannot exclude high grade squamous intraepithelial lesion; LSIL, low-grade squamous intraepithelial lesion; HSIL, high grade squamous intraepithelial lesion; atypical glandular cells (AGC); adenocarcinoma in situ (AIS); Pap, Papanicolaou; CIN, cervical intraepithelial neoplasia; NA, not applicable

*Pearson Chi-square test

**Four cases were inadequate for Pap testing, of which two cases tested positive for hrHPV. Low-grade atypia includes ASC-US, LSIL, and AGC. High-grade atypia includes ASC-H, HSIL, and AIS

Data are expressed in numbers (percentages)

A *p*-value ≤ 0.05 is considered significant

There was a strong statistical association between the presence of EA and the detection of hrHPV, with a significantly higher percentage among hrHPV-positive compared to negative cases (35.7% vs. 4.3%, *p* = 0.001) (Table 2). Moreover, NILM mostly exhibited the absence of hrHPV in 72.4% (42/58) of cases, while the remaining were positive for hrHPV (27.6%, 16/58). Notably, hrHPV infection was encountered in 83.3% (10/12) of atypical Pap results, while 16.7% (2/12) of EA showed undetectable hrHPV. Interestingly, only one case with Pap atypia of the ASC-H type had genital warts (8.3%, 1/12).

In addition, we found a statistically significant predominance of low-grade and high-grade atypia among hrHPV-positive patients compared to negative patients (17.9% and 17.9% vs. 4.3% and 0.0%, respectively) (*p* = < 0.001) (Table 2). Noteworthy, all high-grade atypia cases (including ASC-H, HSIL, and AIS) were exclusively positive for hrHPV detection, meanwhile hrHPV positivity was found in 71.4% (5/7) of low-grade atypia cases (including ASC-US, LSIL, and AGC). Regarding the type of EA, the studied two glandular atypia cases (AGC and AIS) were 100% positive for hrHPV detection, with a statistically significant difference between hrHPV-positive and negative cases (*p* = 0.001).

**Fig. 7:**
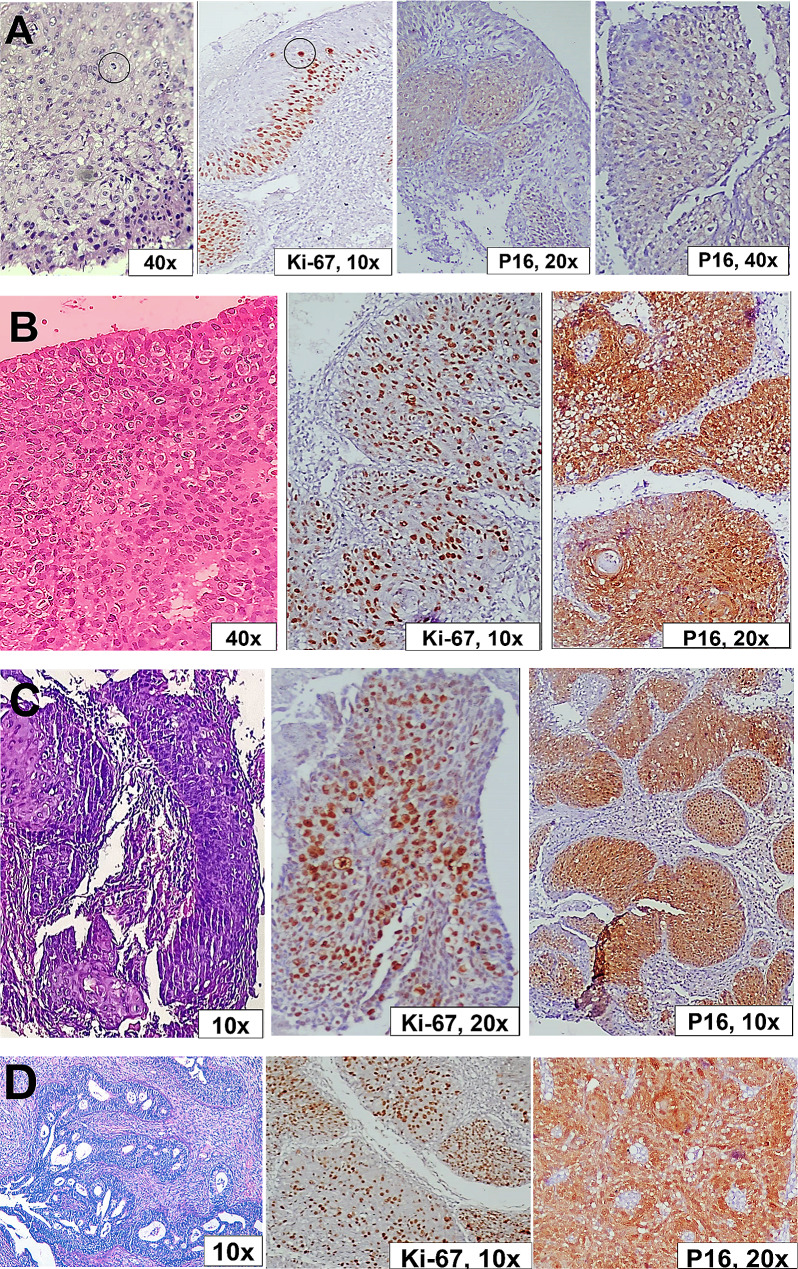
Tissue sections of **A** LSIL (CIN I) Ectocervical epithelium showed increased lower one-third thickness proliferation with detected mid-epithelial mitotic figures (circles) and mild P16 positivity. **B** HSIL (CIN III) The epithelium showed full thickness atypical cellular proliferation and marked P16 positivity. **C** Squamous cell carcinoma (SCC). Surface dysplastic epithelium with infiltrating squamous nests with high proliferation and marked P16 positivity. **D** Adenocarcinoma. Infiltrating cribriform fused irregular glands with high proliferation and marked P16 positivity (H&E stain, Ki-67 and P16 immune stains, 10x, 20x, and 40x original magnifications)

Of the five high-grade atypia cases, only four hrHPV-positive cases underwent colposcopy and tissue biopsy (with one missing patient of the ASC-H type). Two of them were diagnosed as carcinoma (squamous cell carcinoma on top of HSIL and adenocarcinoma on top of AIS), while the other two cases showed cervical intraepithelial neoplasia III (CIN III) (diagnosed as ASC-H by Pap test). The four cases showed strong positive en bloc staining for p16 immune marker (both nuclear and cytoplasmic staining), as well as marked proliferation as displayed by evident Ki-67 positive nuclear staining (Fig. 7). Surprisingly, a total hysterectomy was performed for dysfunctional uterine bleeding in a single case of LSIL. The sections of cervical CIN I were carefully examined, and immunohistochemical staining was employed to emphasize the criteria for HSIL and carcinoma.

As regards the inflammatory environment in Pap testing, most cases showed inflammatory changes (55/74, 74.3%). Marked inflammation was documented in 47.3% (26/55), with noted reactive cellular changes in 49.1% (27/55). Comparing hrHPV positive vs. negative cases, a significant statistical difference was detected (*p* = 0.047), with a higher percentage of marked inflammation among hrHPV-positive cases (46.4%) compared to negative cases (28.3%). Noteworthy, most cases with mild inflammation were negative for hrHPV (22/29, 75.9%).

### High-risk HPV genotype distribution

The most prevalent hrHPV genotype was HPV 16 (25/28, 89.3%). Only one case (3.6%) with the HPV 18 genotype was detected, while the other genotypes were found in two cases (7.1%) as shown in Table 3. All hrHPV-positive cases with NILM and low-grade atypia were infected with HPV 16 (100% each). Among the five cases with high-grade atypia, three were infected with HPV 16 (60%), while the remaining two were infected with other genotypes (40%).

**Table 3:** Distribution of hrHPV genotypes

Parameter *N* (%)	Total *N* = 28 (37.8)	HPV 16 *N* = 25 (89.3)	HPV 18 *N* = 1 (3.6)	Other genotypes *N* = 2 (7.1)
**Age groups**				
˂ 30	8 (28.6)	8 (100)	0 (0.0)	0 (0.0)
30 − 45	17 (60.7)	14 (82.3)	1 (5.9)	2 (11.8)
> 45	3 (10.7)	3 (100)	0 (0.0)	0 (0.0)
Grade of EA** ( ***N*** ** = 26)**				
NILM	16 (61.6)	16 (100)	0 (0.0)	0 (0.0)
Low-grade	5 (19.2)	5 (100)	0 (0.0)	0 (0.0)
High-grade	5 (19.2)	3 (60)	0 (0.0)	2 (40)

*Abbreviations* EA, epithelial atypia; NILM, negative for intraepithelial lesion or malignancy

Data are expressed in numbers (percentages)

**Two cases were excluded due to inadequate Pap testing

### Cervical NOx levels in relation to clinical, cytopathological findings, and HPV infection

**Fig. 8:**
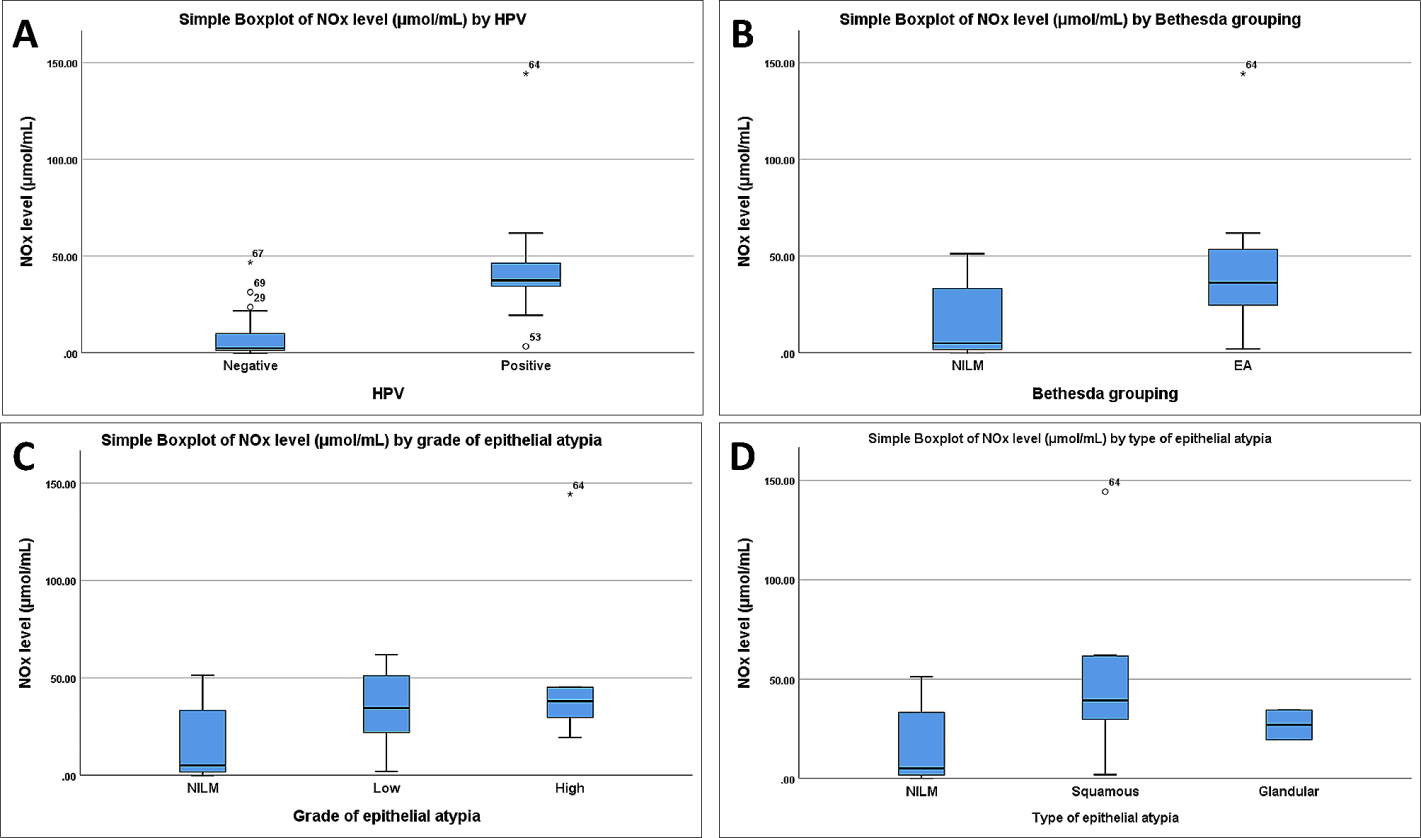
Box-and-whisker plots of cervical NOx levels in the different study groups. **A** Cases with hrHPV-positive and negative results. **B** Cases with negative for intraepithelial lesion or malignancy (NILM) and epithelial atypia (EA). **C** Cases with NILM, high-grade, and low-grade atypia. **D** Cases with NILM, squamous, and, glandular cell types. The solid horizontal lines indicate the median value, the box represents the 25% and 75% interquartile ranges, and the whiskers represent minimum and maximum values

The median total level of cervical NOx was 10.8 µmol/mL (IQR: 1.9–35.7). Different study groups showed different cervical NOx levels as shown in Table 4; Fig. 8. The cervical NOx level showed a statistically significant higher level (*p* = < 0.001) among hrHPV-positive (37.4 µmol/mL, IQR: 34.5–45.8) compared to negative cases (2.3 µmol/mL, IQR: 1.2–9.8) (Fig. 8A). These increased levels were statistically significant as regards each of the studied clinical variables (Table 4). Conversely, the presence of a previous history of genital warts was indifferent between the two groups (*p* = 1). Regarding the use of contraception, the total NOx level was significantly higher among users (35.1 µmol/mL, IQR: 7.9–42.2) compared to non-users (3.6 µmol/mL, IQR:1.6–23.6) (*p* = 0.007) (Table 4).

The total cervical NOx levels in patients with EA were significantly higher (36.2 µmol/mL, IQR: 24.6–53.4) than in patients with NILM (5.0 µmol/mL, IQR: 1.6–33.3) (*p* = 0.001) (Fig. 8B). Moreover, patients with high-grade atypia showed higher NOx levels (38.0 µmol/mL, IQR: 24.6–94.7) compared to those with NILM and low-grade atypia (5.0 µmol/mL, IQR: 1.6–33.3 and 34.5 µmol/mL, IQR: 11.7–61.7, respectively) (*p* = 0.006) (Fig. 8C). The squamous cell type displayed significantly higher NOx levels (39.2 µmol/mL, IQR: 29.7–61.7) compared to the NILM and glandular cell type (5.0 µmol/mL, IQR: 1.6–33.3 and 26.9 µmol/mL, IQR: 19.4–34.5, respectively) (*p* = 0.006) (Fig. 8D) (Table 2).

Comparing hrHPV positive and negative cases, a significant statistical association was found among all the studied cytological parameters (Table 4). Despite NOx level among hrHPV-positive cases with low-grade atypia (40.4 µmol/mL, IQR: 33.3‒61.8) was higher than those with NILM (36.2 µmol/mL, IQR: 35.7‒44.0) and high-grade atypia (38.0 µmol/mL, IQR: 24.6‒94.7), the difference was insignificant (*p* = 0.771).

**Table 4:** Cervical NOx levels among hrHPV-positive and negative cases concerning clinical and cytopathological findings

Clinical variables	Total*	hrHPV-positive	hrHPV-negative	*p*-value
**Cervical NOx level**	10.8 (1.9–35.7)	37.4 (34.5–45.8)	2.3 (1.2–9.8)	**< 0.001**
**Clinical characteristics**				
**Age groups**				
˂ 30	10.6 (1.5–35.1)	35.7 (33.9–36.8)	1.8 (< 1–6.0)	< 0.001
30 − 45	14.7 (2.6–38.0)	38.0 (35.7–46.4)	2.9 (1.7–12.2)	**< 0.001**
> 45	4.7 (1.4–16.5)	40.4 (35.1–51.2)	2.8 (1.2–6.8)	**0.003**
**Gravidity**				
Nulligravida	6.6 (1.9–34.5)	36.8 (35.7–46.4	2.3 (1.7– 8.5)	**< 0.001**
Multigravida	13.9 (1.9–36.8)	38.0 (31.7–42.8	2.4 (1.2– 7.6)	**< 0.001**
**Parity**				
Nulliparous	6.6 (1.9–34.5)	36.8 (35.7–46.4)	2.3 (1.7–8.5)	**< 0.001**
Parous	13.9 (1.9–36.8)	38.0 (31.7–42.8)	2.4 (1.2–7.6)	**< 0.001**
**Contraception**				
Yes	35.1^a^ (7.9–42.2)	38.0 (35.7–46.4)	2.4 (1.1–2.6)	**< 0.001**
No	3.6^a^ (1.6–23.6)	35.7 (34.5–40.4)	2.1 (1.2–6.8)	**< 0.001**
**Contraceptive method**				
None	3.5 (1.8–21.7)	35.7 (33.9–40.4)	2.1 (1.2–6.8)	**< 0.001**
Oral	47 (46.4–47.5)	47.0 (46.4–47.6)	–	NA
IUD	34.5 (7.8–38)	38.0 (35.1–44.0)	1.7 (< 1–7.8)	**< 0.001**
Others	38 (20.3–39.2)	39.2 (38.0–40.4)	–	NA
**Sexual partner**				
Yes	12.8 (2.4–35.7)	37.4 (34.5–45.2)	2.6 (1.8─11.4)	**< 0.001**
No	4.7 (1.4–33.3)	36.8 (35.7–45.2)	1.9 (< 1–5.7)	**< 0.001**
**History of genital warts**				
Yes	38.2 (17.5–49.0)	40.4 (29.7–51.2)	26.1 (5.4–46.7)	1
No	10.0 (1.9–35.7)	37.4 (34.5–45.2)	2.2 (1.2–8.3)	**< 0.001**
**History of abnormal PAP smear**				
Yes	30.5 (18.0–44.2)	35.7 (24.6–51.6)	23.9 (9.0–39.0)	0.486
No	6.6 (1.9–35.7)	37.4 (35.0–45.8)	2.2 (1.2–6.3)	**< 0.001**
**Cytological findings**				
**Bethesda grouping**				
NILM	5.0^b^ (1.6–33.3)	36.2 (35.7‒44.0)	2.4 (1.2‒8.3)	**< 0.001**
EA	36.2^b^ (24.6–53.4)	39.2 (33.3‒53.4)	6.8 (1.9‒11.7)	**0.03**
**Grade of EA**				
NILM	5.0^c^ (1.6–33.3)	36.2^g^ (35.7‒44.0)	2.4 (1.2‒8.3)	**< 0.001**
Low-grade	34.5^c^ (11.7–61.7)	40.4^g^ (33.3‒61.8)	6.8 (1.9‒11.7)	**0.053**
High-grade	38.0^c^ (24.6–94.7)	38.0^g^ (24.6‒94.7)	–	NA
**Type of EA**				
NILM	5.0^d^ (1.6–33.3)	36.2 (35.7‒44.0)	2.4 (1.2‒8.3)	**< 0.001**
Squamous	39.2^d^ (29.7–61.7)	42.8 (35.1‒61.8)	6.8 (1.9‒11.7)	**0.044**
Glandular	26.9^d^ (19.4–34.5)	26.9 (19.4‒34.5)	–	NA
**Biopsy results**				
Dysplasia (CIN III)	45.7 (29.7–61.7)	45.7 (29.7–61.7)	–	NA
Carcinoma	81.9 (19.4–144.3)	81.9 (19.4–144.3)	–	NA
**Inflammation**				
Yes	11.7^e^ (1.9–35.7)	37.4 (33.3–45.8)	2.6 (1.4–11.5)	**< 0.001**
No	4.7^e^ (1.8–35.7)	36.8 (35.1–47.1)	1.9 (< 1–2.8)	**< 0.001**
**Inflammation**				
Mild	3.3 (1.4–28.5)	38.0 (32.1–44.6)	2.0 (1.1–12.8)	**< 0.001**
Marked	20.6 (5.4–36.8)	36.8 (34.5–45.2)	5.4 (2.4–11.4)	**< 0.001**
**Reactive cells**				
Yes	21.7^f^ (2.1–37.4)	38.0 (34.5‒46.4)	2.1 (1.2–11.7)	**< 0.001**
No	5.4^f^ (1.8–35.0)	35.7 (35.1–42.8)	2.3 (1.3–6.6)	**< 0.001**

*Abbreviations* IUD, intrauterine device; NILM, negative for intraepithelial lesion or malignancy; EA, epithelial atypia; CIN, cervical intraepithelial neoplasia; NA, not applicable

^a^*p* = 0.007. ^b^*p* = 0.001. ^c^*p* = 0.006. ^d^*p* = 0.006. ^e^*p* = 0.748. ^f^*p* = 0.191. ^g^*p* = 0.771

*All statistical comparisons were calculated using Mann-Whitney *U* test, except the grade and type of EA where the Kruskal-Wallis test was used instead

Cervical NOx levels are measured in µmol/mL

Data are expressed in median (interquartile range)

A *p*-value ≤ 0.05 is considered significant

**Fig. 9:**
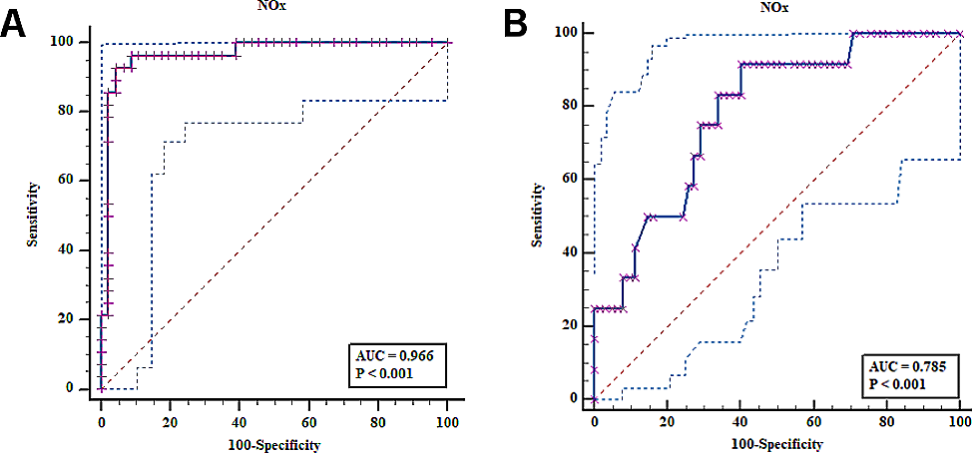
ROC curves of cervical NOx levels for **A** hrHPV-positive *versus* negative cases. **B** Patients with normal *versus* abnormal Pap results

The ROC curve analysis for cervical NOx level in the hrHPV infected vs. non-infected patients and in those with normal vs. abnormal Pap test results are shown in Fig. 9. Cervical NOx level had good diagnostic accuracy for differentiating patients infected with hrHPV from non-infected patients (AUC = 0.966) (Fig. 9A), and women with atypia from those having normal Pap test results (AUC = 0.785) (Fig. 9B). According to the proposed cut-off values, the sensitivity, specificity, PPV, and NPV of the cervical NOx level were calculated (Table 5).

**Table 5:** Results of ROC curve analysis of cervical NOx levels in the different study groups

Test variable	Optimal Cut-off	AUC*	Sensitivity (%)	Specificity (%)	PPV* (%)	NPV* (%)	*p*-value	95% CI
**hrHPV-positive vs. negative cases**								
Nitric oxide (µmol/mL)	> 23.61	0.966	92.86	95.65	92.9	95.7	**< 0.0001**	0.895–0.994
**Pap normal vs.** **abnormal results**								
Nitric oxide (µmol/mL)	> 11.35	0.785	91.67	59.68	30.6	97.4	**< 0.0001**	0.674–0.872

*Abbreviations AUC, area under the curve; PPV, positive predictive value; NPV, negative predictive value

## Discussion

Most HPV infections are asymptomatic and resolve spontaneously without treatment. However, certain HPV genotypes, such as HPV 16 and 18, can result in persistent infections [17]. The HPV information center’s latest estimates in Egypt stated that every year 1,320 females are diagnosed with cancer cervix, with a reported 744 deaths annually [18]. Seventy-four patients seeking gynecological consultation in Gynecology and Obstetrics outpatient clinics at Cairo University hospitals were enrolled in this study, with a mean age of 38.7 ± 10.9 years. High-risk HPV was detected in 37.8% of the studied cases. This rate was near to Prakash et al. who detected HPV 16/18 in 33.6% of their studied cohort [19]. By contrast, Abdel Aziz et al. [20] and Rahkola et al. [11] reported higher rates, with 64% and 53.4% of their respective cohorts testing positive for hrHPV. A lower rate was reported in Egypt (16.1%) [21], Iraq (19%) [22], and China (15.6%) [23]. The differences in reported hrHPV prevalence rates across different studies can be attributed to the various geographic distribution, the transient nature of HPV infections, variations in the tests used to measure prevalence, and lower rates in countries with successful vaccination programs.

In our study, the hrHPV was mostly encountered in the age group 30‒45 years, accounting for 60.7% of cases, while a notable low rate (10.7%) was detected in those over 45 years. This was consistent with a previous Egyptian study showing that most affected cases were in the age group 35‒45 years. Women within this age group who tested positive for hrHPV are likely to experience a long-standing persistent infection, which highlights the significance of promoting awareness among women within this age group [24]. Furthermore, we reported a statistically significant relationship between hrHPV infection and the use of contraceptives (*p* = 0.002), being detected in 39.3% of hrHPV-positive cases. Ibrahim et al. [25] have shown no association between hrHPV infection and the use of contraception. Our results point to the probability that the prolonged use of IUDs could potentially increase the risk of HPV infection due to localized irritation, disruption of the cervical barrier, and modulation of the immune response. However, to attain a complete understanding of this association, it is necessary to conduct broader additional research.

It is worth mentioning that low-risk HPV types 6 and 11 usually account for 90% of genital warts, whereas most cervical cancers are attributed to high-risk HPV types 16 and 18 [26]. In our research, we exclusively examined high-risk genotypes and found four cases (5.4%) with a history of previous genital warts, among them two cases showed the presence of HPV genotype 16. According to Bruni et al., it was reported that 4.4‒36.7% of women with genital warts tested positive for hrHPV [27]. Interestingly, we detected only one case with EA of the ASC-H type (8.3%, 1/12) that had genital warts, suggesting a potential link between the presence of genital warts and cervical neoplasia associated with hrHPV infection.

Our study revealed that Pap smear was inadequate in four (5.4%) cases. Epithelial atypia was found in 17.1% (12/70) of cases, while 82.9% (58/70) fell under the NILM category. Our finding is higher than that of a previous Egyptian national cervical cancer screening project in 2007; as EA was identified in 8% of women [28]. In addition, Elazab et al. [24] found that 9.7% (97/1000) of women attending Egyptian public healthcare hospitals exhibited different degrees of CIN. The dissimilarity in results can be attributed to the different inclusion criteria, as their study focused exclusively on asymptomatic women without a history of recurrent vaginal or cervical infections, as well as the different used Pap test types. These findings potentially indicate an emerging trend of increased CIN cases in Egypt, particularly considering the information provided by the World Health Organization (WHO) stating that less than 10% of Egyptian women have undergone cervical cancer screening in the past five years [29]. This highlights the critical need for the implementation of a cervical cancer screening program in our country.

In this work, NILM exhibited the absence of hrHPV in most cases (42/58, 72.4%), whereas hrHPV was found in 27.6% (16/58) of cases with NILM pap results. This was near to studies conducted by Prakash et al. [19] and Youssef et al. [21] who detected hrHPV in 20.8% and 18.2% of infections among cytologically normal women. A much lower rate was reported by Abdel Aziz et al. [20] who found that 3.8% (4/106) of those having normal cytology showed hrHPV positivity. Therefore, hrHPV DNA detection in clinically and cytologically normal subjects is valuable for determining those at risk of developing intraepithelial lesions. This highlights the limitations of relying solely on cytology for detecting women at risk of cervical cancer, as hrHPV DNA testing provides a standardized approach with superior sensitivity in detecting CIN III lesions compared to cytology [30]; however, the significance of viral load remains to be elucidated. Thrall et al. [31] examined a debatable concern that often arises among cytologists and practitioners: are Pap tests that are interpreted as NILM but show positive results for hrHPV truly devoid of dysplasia evidence, or was dysplasia overlooked during screening? They checked hrHPV testing in females aged 30 years or older with NILM Pap test results and found that 5.4% (146/2,719) of cases were hrHPV-positive, with approximately 75% of them spontaneously resolved within about 3 years.

In our study, hrHPV DNA was detected in 83.3% (10/12) of those with atypical pap results, while 16.7% (2/12) of EA showed undetectable hrHPV. There was a strong relationship between EA in pap testing and hrHPV detection (*p* = 0.001). Likewise, previous studies reported a significant association between the presence of hrHPV and abnormal cytology [20, 25]. The high-grade atypia (including ASC-H, HSIL, and AIS) was exclusively positive for hrHPV, whereas 71.4% of low-grade atypia (including ASC-US, LSIL, and AGC) were positive for hrHPV. The studied two glandular atypia cases were positive for hrHPV, with a strongly significant relationship (*p* = 0.001). According to our results, it appears that HPV infection increases the risk of cervical neoplasia as lesions progress to higher grades. This was in concordance with Oh et al. [32] who documented that HPV positivity was significantly associated with HSIL or higher grades. They detected hrHPV in 10.5% of ASC-US, 25.0% of LSIL, 78.8% of HSIL, and all cases of SCC. An additional study reported HPV 16/18 positivity in 44% and 66.7% among cases of low and high-grade atypia, respectively [19]. In Mexico, hrHPV was reported in 40.7% of LSIL, 56.5% of HSIL, and 81.6% of cervical cancer cases [33].

In our investigation, most cases (74.3%) exhibited inflammatory changes, and we observed a significant association between marked inflammation and hrHPV infection (*p* = 0.047). Additionally, 75.9% (22/29) of cases with mild inflammatory processes tested negative for hrHPV. Similarly, a previous Egyptian study found that 63% of their studied women had inflammatory changes, and HPV mostly 16/18 was commonly associated with mixed infections (*p* < 0.001) [28]. According to a prior study, it has been suggested that hrHPV might induce the activation of NF-𝜅B p65 and inducible nitric oxide synthase (iNOS) pathways, triggering inflammation [34]. Furthermore, excess inflammatory cells may obscure the interpretation of cellular changes associated with HPV infections [15].

According to global estimates, HPV 16 and 18 are responsible for approximately 70% of cervical cancers [35]. Our research found that HPV 16 was the most common genotype (25/28, 89.3%), with a low prevalence of HPV 18 (3.6%). These findings were in line with previous studies conducted in Egypt [21, 25] and Iraq [22]. The discrepancy in results among different studies was attributed to the variations in the sensitivity of different HPV detection methods [36]. Moreover, our study found that all cases of normal Pap test results were of the HPV 16 genotype (100%), which agrees with a previous investigation [37]. Our findings highlight the importance of performing HPV genotyping in females with normal cytological results and those who tested positive for hrHPV types should undergo HPV retesting at one-year intervals to exclude viral persistence. On the other hand, we detected two cases with high-grade atypia infected with genotypes other than 16 and 18 that showed negative results for NILM, indicating a high pathogenic potential associated with these genotypes.

To the best of our knowledge, this is the first Egyptian study to examine the cervical fluid NOx levels in relation to Pap smear and HPV infections among a group of women using in-vitro diagnostic testing. In our study, the cervical NOx level was significantly higher (*p* = < 0.001) among hrHPV-positive (37.4 µmol/mL, IQR: 34.5–45.8) compared to negative cases (2.3 µmol/mL, IQR: 1.2–9.8). Consistent with our results, earlier studies demonstrated a notable increase in cervical NO release among individuals infected with hrHPV compared to those who were uninfected or had a low-risk HPV infection. This observation indicates a strong association between cervical NOx levels and hrHPV infection [11, 12]. Moreover, Li et al. [34] confirmed the elevated expression of cervical iNOS of hrHPV-infected patients, with a subsequent substantial increase in the cervical NOx release. It has been shown that HPV infection can trigger the activation of many inflammatory cells in the infected cervix, promoting the release of cytokines that can trigger NO synthesis in cervical and inflammatory cells through iNOS activation [10]. In our study, the elevated NOx levels were unaffected by several factors, including age, gravidity, parity, contraceptive method, or the existence of a sexual partner, a finding supported by our study as well as Rahkola et al. [11]

In our work, we found that cervical NOx levels in patients with EA were significantly higher (36.2 µmol/mL, IQR: 24.6–53.4) compared to patients with NILM (5.0 µmol/mL, IQR: 1.6–33.3) (*p* = 0.001). Similarly, Rahkola et al. observed that females with abnormal cytology had higher NOx levels (median 22.5 mmol/L) compared to those with normal cytology (median 11.0 mmol/L) [38]. Moreover, we found that patients with high-grade atypia showed significantly higher NOx levels (38.0 µmol/mL, IQR: 24.6–94.7) compared to those with NILM (5.0 µmol/mL, IQR: 1.6–33.3) and low-grade atypia (34.5 µmol/mL, IQR: 11.7–61.7) (*p* = 0.006). On the contrary, Giannella et al. [39] observed that high-grade CIN was associated with decreased NOx levels compared to low-grade CIN. The discrepancy in results could be linked to the fact that Giannella and coworkers had excluded local infections as confounding factors in their study, and their study design was specifically focused on women who had abnormal pap smears and had CIN cervical biopsy results.

Regarding NOx levels among hrHPV-positive cases, low-grade atypia (40.4 µmol/mL, IQR: 33.3‒61.8) displayed higher NOx levels than NILM (36.2 µmol/mL, IQR: 35.7‒44.0) and high-grade atypia (38.0 µmol/mL, IQR: 24.6‒94.7) (*p* = 0.771). Our findings agree with Rahkola et al. who found that hrHPV-positive cases with low-grade atypia (57.41 mol/L) were significantly associated with higher NOx levels compared to high-grade atypia (28.91 mol/L) (*p* = 0.001) [12]. They postulated that an initial robust immune response triggered by HPV infection results in elevated cervical NOx production. Subsequently, as women progress towards dysplasia, their immune system weakens, leading to decreased cervical NOx levels [12].

In our study, ROC curve analysis indicated that the cervical NOx cut-off values of > 23.61 µmol/mL (AUC = 0.966) and > 11.35 µmol/mL (AUC = 0.785) exhibited the highest sensitivity and specificity for prediction of hrHPV infection and EA, respectively. In a study by Rahkola et al. [12] the 75th percentile NOx level (87.0 µmol/L) was found to be a significant predictor of hrHPV persistence at 12 months when compared to hrHPV-negative women (Odds ratio (OR) = 4.1; 95% CI, 1.3–13.1), however, the 75th percentile NOx level failed to predict cytological changes (OR = 1.9; 95% CI: 0.7–5.4). In contrast, Giannella et al. [39] found that decreased cervical NOx levels (≤ 99.9 µmol/L) may be used for the prediction of high-grade CIN (AUC = 0.766). Nevertheless, their study was limited by the lack of inclusion of HPV status, primarily due to the availability of HPV status information for only a small number of women.

Our study had some limitations. First, we had to exclude four cases from analysis due to inadequate Pap samples, however, we carefully analyzed the remaining data to ensure the reliability and accuracy of the cytopathological results. Second, there was a failure to conduct a colposcopic examination and perform a tissue biopsy, if necessary, for one patient diagnosed with ASC-H. Third, the inability to determine the genotype of two hrHPV cases. Nevertheless, it is crucial to highlight that all the evaluated hrHPV genotypes are highly pathogenic and have the potential to cause cervical cancer [40]. In contrast, to ensure accurate data collection and elimination of the variation between different laboratories, we tested all samples with a standardized DNA test in a single laboratory, contributing to the strength of our study.

## Conclusions

The current study showed that hrHPV infection is highly prevalent in our hospital, with the predominance of genotype HPV 16. Our findings found a strong association between the presence of hrHPV and the development of EA that highlights the need for targeted treatment approaches, follow-up of patients, as well as implementing the cervical screening program using combined HPV tests and conventional cytology. The results of this work revealed an association between elevated cervical NOx levels with hrHPV infection and high-grade atypia suggesting the potential of using NOx levels as a non-invasive biomarker for predicting the presence of hrHPV and abnormal cytological changes. However, further multi-center studies involving a larger sample size are needed to validate and strengthen this finding.

## Data Availability

The authors confirm that all datasets generated and/or analyzed during this study are presented in the main manuscript and are available from the corresponding author upon reasonable request.

## Abbreviations

ACI: Adenocarcinoma in situ
AGC: Atypical glandular cells
ASC-H: Atypical squamous cells-cannot exclude a high-grade squamous intraepithelial lesion
ASC-US: Atypical squamous cells of undetermined significance
CIN III: Cervical intraepithelial neoplasia III
EA: Epithelial atypia
HrHPV: High-risk human papillomavirus
HSIL: High-grade squamous intraepithelial lesion
iNOS: Nitric oxide synthase
LSIL: Low-grade squamous intraepithelial lesion
NILM: Negative for intraepithelial lesion or malignancy
NO: Nitric oxide
OS: Oxidative stress
Pap: Papanicolaou
PCR: Polymerase chain reaction
RONS: Reactive oxygen and nitrogen species
TBS: The Bethesda System
